# Control of redox potential in a novel continuous bioelectrochemical system led to remarkable metabolic and energetic responses of *Clostridium pasteurianum* grown on glycerol

**DOI:** 10.1186/s12934-022-01902-5

**Published:** 2022-09-01

**Authors:** Philipp Arbter, Niklas Widderich, Tyll Utesch, Yaeseong Hong, An-Ping Zeng

**Affiliations:** 1 Institute of Bioprocess and Biosystems Engineering, Hamburg University of Technology, Denickestraße 15, 21073 Hamburg, Germany; 2 Present Address: Center of Synthetic Biology and Integrated Bioengineering, School of Engineering, Westlake University, Hangzhou, 310024 Zhejiang China

**Keywords:** BES, ORP, *Clostridium pasteurianum*, Continuous fermentation, Redox metabolism, Regulation analysis, Symbolic metabolic control analysis

## Abstract

**Background:**

Electro-fermentation (EF) is an emerging tool for bioprocess intensification. Benefits are especially expected for bioprocesses in which the cells are enabled to exchange electrons with electrode surfaces directly. It has also been demonstrated that the use of electrical energy in BES can increase bioprocess performance by indirect secondary effects. In this case, the electricity is used to alter process parameters and indirectly activate desired pathways. In many bioprocesses, oxidation-reduction potential (ORP) is a crucial process parameter. While *C. pasteurianum* fermentation of glycerol has been shown to be significantly influenced electrochemically, the underlying mechanisms are not clear. To this end, we developed a system for the electrochemical control of ORP in continuous culture to quantitatively study the effects of ORP alteration on *C. pasteurianum* by metabolic flux analysis (MFA), targeted metabolomics, sensitivity and regulation analysis.

**Results:**

In the ORP range of −462 mV to −250 mV, the developed algorithm enabled a stable anodic electrochemical control of ORP at desired set-points and a fixed dilution rate of 0.1 h^−1^. An overall increase of 57% in the molar yield for 1,3-propanediol was observed by an ORP increase from −462 to −250 mV. MFA suggests that *C. pasteurianum* possesses and uses cellular energy generation mechanisms in addition to substrate-level phosphorylation. The sensitivity analysis showed that ORP exerted its strongest impact on the reaction of pyruvate-ferredoxin-oxidoreductase. The regulation analysis revealed that this influence is mainly of a direct nature. Hence, the observed metabolic shifts are primarily caused by direct inhibition of the enzyme upon electrochemical production of oxygen. A similar effect was observed for the enzyme pyruvate-formate-lyase at elevated ORP levels.

**Conclusions:**

The results show that electrochemical ORP alteration is a suitable tool to steer the metabolism of *C. pasteurianum* and increase product yield for 1,3-propanediol in continuous culture. The approach might also be useful for application with further anaerobic or anoxic bioprocesses. However, to maximize the technique's efficiency, it is essential to understand the chemistry behind the ORP change and how the microbial system responds to it by transmitted or direct effects.

**Supplementary Information:**

The online version contains supplementary material available at 10.1186/s12934-022-01902-5.

## Introduction

Electrobiotechnology is a promising technology at the interface of biotechnology and electrochemistry. In this context, an especially high potential is expected for bioprocesses in which the cells are enabled to directly harvest electricity-derived electrons in bioelectrochemical systems (BES) [1]. But experiments have also demonstrated that using electrical energy in BES can increase the performance of fermentation processes even when cells cannot directly exchange electrons with electrode materials [2]. The electricity is then used to alter process parameters and indirectly activate desired pathways. Here, the most essential process parameter is the oxidation-reduction potential (ORP), which can easily be tracked online by redox probes (usually with Ag/AgCl electrodes) during cultivation. It is well-known that ORP, sometimes also named E_h_ in literature [3], is one of the key parameters in many biotechnological processes. Moreover, it has been shown to affect microbes on different metabolic levels. This concerns gene expression [4, 5] but also signal sensing, signal transduction, and regulation [6, 7].

**Fig. 1:**
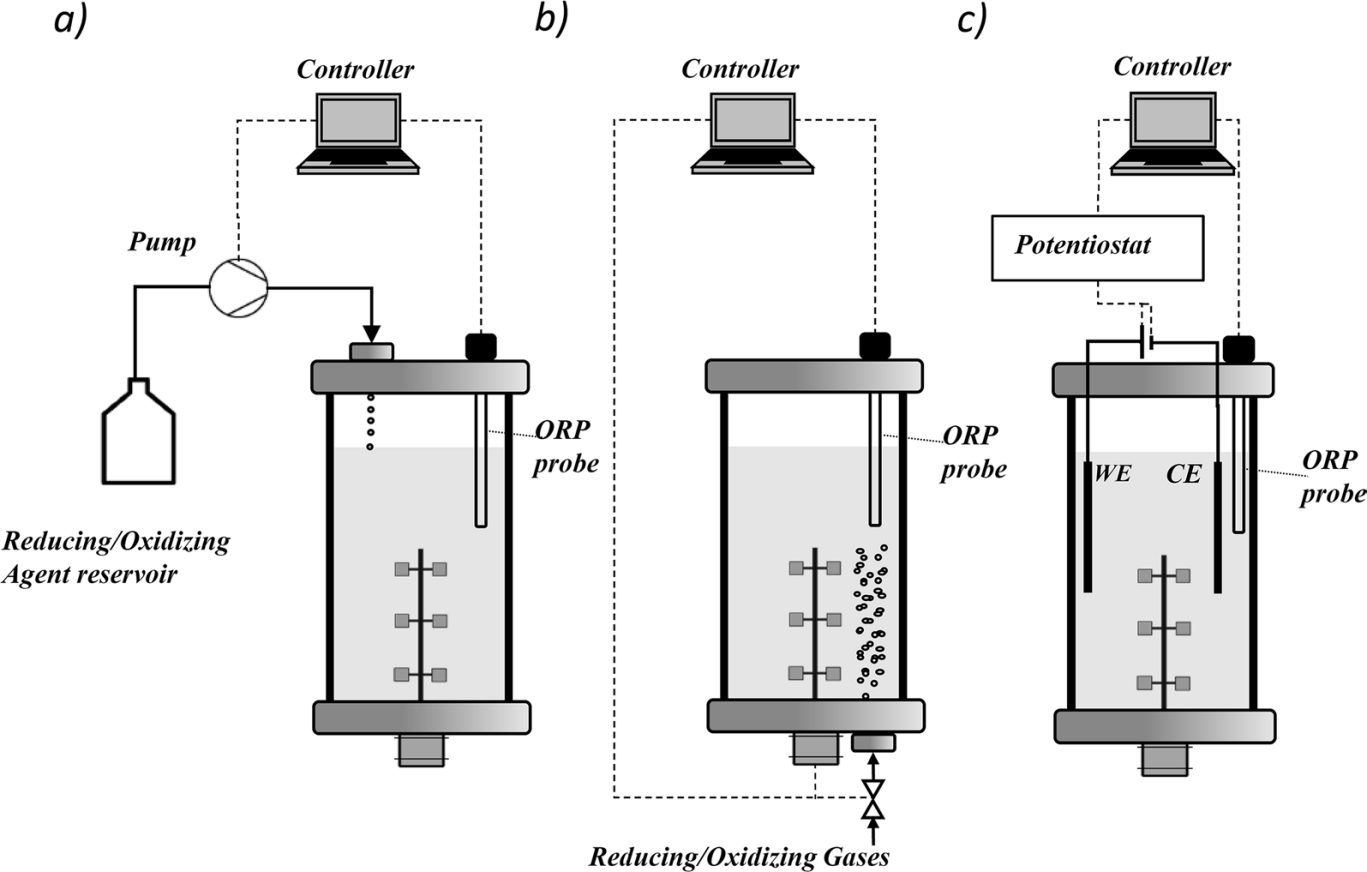
Different strategies for ORP control in fermentation processes. **a** Addition of liquid reducing/oxidizing agents. **b** Sparging with reducing/oxidizing gases (control of aeration rate and agitation). **c** Application of electrical energy in a BES. WE = working electrode; CE = counter electrode

Different strategies for alteration and control of ORP have been developed and applied [8]. Figure 1 gives an overview of the different approaches. One often chosen method is the addition of highly-oxidizing or reducing agents into the fermentation medium (Fig. 1a), such as ferricyanides [9], sulfides [10], or redox mediators [11, 12]. Although ORP can be controlled sufficiently by this strategy in most cases, adding chemicals also has some disadvantages. One major drawback is that adding these (often toxic) compounds in non-negligible quantities alters the fermentation medium's composition. Here, it might occur that the study results do not primarily reflect the metabolic response to the ORP parameter but the reaction of cellular metabolism to the added chemical. Hence, a better alternative option to control the ORP is by sparging gases (Fig. 1b), such as oxygen, hydrogen, nitrogen, or helium [13–15]. To control gas solubility and accordingly the ORP, agitation [16, 17] or the aeration rate [18] are used as controller inputs. The use of gases has the advantage that they usually have low solubility, are (mostly) not toxic to the cells, and do not accumulate in the liquid phase of the reaction system. However, one practical disadvantage of the ORP control with gas sparging is that the control algorithms are more difficult to implement. Furthermore,the desired set-points are more challenging to achieve and maintain, especially when the related gas is also consumed or produced by the microbes and the physicochemical properties of the fermentation broth change during the time of cultivation. Moreover, gassing can interfere with the cultivation process and result in the stripping of CO_2_ or other volatile components.

A third option to control ORP in fermentation processes is using electrical energy in BES (Fig. 1c). In literature, only a few examples exist of energy input used for ORP alteration. Almost all of the conducted studies so far used cathodic current to lower or control the ORP in anaerobic or microaerobic processes [19–23]. Only two dealt with ORP manipulation under aerobic or microaerobic conditions [24, 25]. None of the cited studies aimed to control ORP at desired levels at fixed dilution rates in continuous culture, which has decisive benefits for kinetic and physiological investigations. Hence, in this study, we aimed to develop an electrochemical ORP control with the help of a previously developed "All-in-One" electrode [26] in a continuous BES. *Clostridium pasteurianum* is a promising candidate for industrial production of the valuable chemicals 1,3-propanediol (PDO) and n-butanol [27, 28]. Previous work has shown that *C. pasteurianum* cannot be considered electroactive but that its metabolism is still affected by the application of electrical energy [29, 30]. Depending on the conditions, BES cultivations favour PDO or n-butanol production, but the mechanisms are not well understood. Therefore, this work aimed to gain more knowledge on the underlying metabolic shifts with the developed continuous system. To gain quantitative and reliable insights, targeted metabolomic anaylsis and metabolic flux analysis (MFA) are used. The derived steady-state metabolite concentrations and fluxes are then used to determine the underlying control pattern by using metabolic control analysis (MCA) and to quantitatively study the effect of the ORP change by sensitivity and regulation analysis.

## Results

### Implementation of an electrochemical ORP control

For the continuous cultivations, cells were initially grown to steady state without power application. Then, the first attempts to control ORP used a constant cathodic current of −100 mA with a simple on/off controller. Here, the first finding was that the ORP could, in contrast to the results obtained in fed-batch fermentations [29], not be reduced further at the fixed dilution rate of 0.1 h^−1^ in the BES. This remained the case even when the current (and electrochemical hydrogen production rate) was increased up to −1.0 A. Also, even when the system was operated overnight with the constant application of only low currents (cathodic or anodic were tested both), washout of the cells was observed. Here, it is assumed that the electrochemical adsorption of charged trace elements may have prevented the cells from achieving the required growth rate at the set dilution rate.

**Fig. 2:**
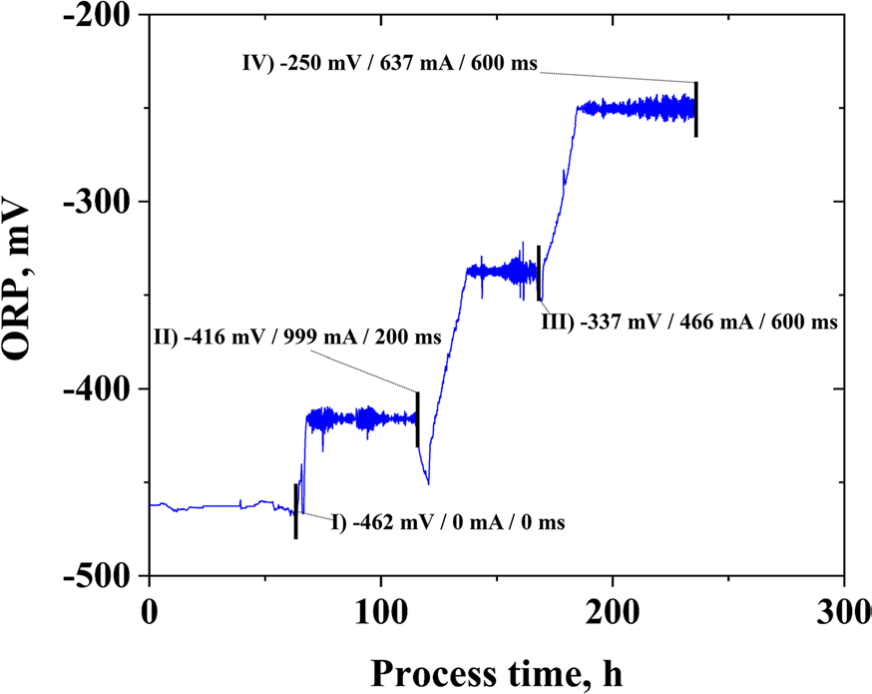
Online ORP values (in mV) during the continuous electrochemically ORP-controlled cultivation of *C. pasteurianum* in a BES at D = 0.1 h^−1^. Captures indicate the following: Number of steady state (I-IV); set-points for the electrochemical ORP control (in mV) / applied current (in mA) / pulsing time (in ms). A detailed description of the control mechanism is given in the text. The electrochemical pulsing was interrupted by 100 ms without current application. Black blocks indicate time points of fast-sampling and changing of controller set-points

In the next step, to avoid cell washout in subsequent experiments, a pulse control algorithm was implemented for the ORP control. The controller was a simple manually tuned PI-controller (implemented via Labview), using the ORP online value as the input variable and the applied current as output. The pulsing time was 100 ms, followed by 100 ms without the application of electricity. Here, it turned out that the initial duration of 100 ms could not increase the ORP sufficiently, even at the used potentiostat's maximal current (1.0 A). In this case, when no increase of ORP was observed after 3 h, the pulsing time was manually increased stepwise by 100 ms. The period without electricity was kept constant at 100 ms. This strategy made it possible to maintain the ORP at the desired set-point at a dilution rate of 0.1 h^−1^ (+−10 mV). Overall, four different ORP levels at dilution rates of 0.1 h^−1^ were achieved and evaluated, while the first steady state was without the application of electrical energy. The online ORP values for the tested conditions, including the final controller output values, are shown in Fig. 2. At each steady state, which was assumed to be reached after a minimum of five hydraulic retention times (and verified by an unchanging off-gas composition), fast sampling and filtration for metabolomics analysis were conducted, and four samples in 15 min intervals were taken for analysis of extracellular metabolites. From the latter, rates, yields, and balances were calculated. The determined rates and variances were then used for MFA and subsequently for sensitivity and regulation analysis.

### Influence of different ORP levels on product formation and yields

Table 1 shows the concentrations of extracellular metabolites at each steady state. One can see that the steady-state substrate concentration consistently increased with a more positive ORP. Hence, overall substrate uptake (per L culture volume) decreased. Decreased absolute steady-state concentrations were also observed for biomass and the primary products PDO and n-butanol. In conclusion, the macroscopic data show that ORP strongly affects cell growth and cellular metabolism. *C. pasteurianum* converts less substrate at a more positive ORP, and the cell concentration decreases drastically. This might essentially be contributed to oxygen production by the electrode when running in anodic mode to control the ORP. Already at −416 mV 4% oxygen were detected in the off-gas. At −337 mV it were 6%. At −250 mV, the ' gas production could not be measured since the off-gas stream was lower than the minimum required of the used MS (5 mL min^−1^). Here, estimated and balanced CO_2_ and H_2_ production rates for calculating carbon and electron recoveries were obtained from the MFA. In general, oxygen negatively influences *clostridia*'s growth [31], and oxygen-induced stress appears to be the primary reason for the overall decrease in product and cell concentrations.

**Table 1:** Extracellular concentrations and standard deviation (in g L^−1^) of glycerol (Gly), 1,3-propanediol (PDO), ethanol (Eth), n-butanol (BuOH), lactate (Lac), formate (Form), acetate (Ace), butyrate (Buty) and dry biomass (BM) at steady-state conditions during the continuous ORP controlled-cultivation of *C. pasteurianum* grown on glycerol (D = 0.1 h^−1^ and ${c}_{s,in}$ = 36 g L^−1^)

ORP, mV	Gly, g L^−1^	PDO, g L^−1^	Eth, g L^−1^	BuOH, g L^−1^	Lac, g L^−1^	Form, g L^−1^	Ace, g L^−1^	Buty, g L^−1^	BM, g L^−1^
−462	7.65 ± 0.11	3.64 ± 0.09	0.39 ± 0.08	6.16 ± 0.11	0.00 ± 0.00	0.36 ± 0.02	0.25 ± 0.01	1.45 ± 0.03	2.09 ± 0.25
−416	13.73 ± 0.18	2.47 ± 0.05	0.37 ± 0.05	4.88 ± 0.18	0.63 ± 0.01	0.65 ± 0.02	0.28 ± 0.01	0.25 ± 0.01	1.59 ± 0.14
−337	22.75 ± 0.35	2.2 ± 0.11	0.48 ± 0.14	2.14 ± 0.05	1.13 ± 0.03	0.48 ± 0.02	0.05 ± 0.1	0.32 ± 0.03	0.98 ± 0.17
−250	31.12 ± 0.89	1.17 ± 0.09	0.39 ± 0.08	0.46 ± 0.05	1.21 ± 0.18	0.00 ± 0.00	0.00 ± 0.00	0.00 ± 0.00	0.40 ± 0.06

Table 2 shows the calculated molar yields and recoveries for the experimental data from the continuous fermentation. Even though overall substrate consumption and product formation generally declined with the ORP increase, the molar yields for some products could be improved by the ORP alteration. Here, it is remarkable that the PDO yield could be increased from 149 mmol mol^−1^ (at −462 mV) to a maximum of 234 mmol mol^−1^ (−250 mV), equalling an improvement of 57%. Also, lactate yield and concentration increased significantly with a more positive ORP, while butanol yields decreased. The carbon and electron recoveries for the obtained data are generally unsatisfactory. Even when no electricity was used (at −462 mV), the carbon recovery yielded only 89%. Here, one explanation might be that the low growth rate stressed the cells and triggered the formation of product(s) not tracked by the analytical method. However, no unknown peak could be found in the HPLC chromatograms.

**Table 2:** Molar yields and standard deviation (in mmol mol^−1^) of 1,3-propanediol (PDO), ethanol (Eth), n-butanol (BuOH), lactate (Lac), formate (Form), acetate (Ace), butyrate (Buty) and dry biomass (BM) plus macroscopic carbon (C_R_) and electron recoveries (R_H_) at steady-state conditions during the continuous ORP-controlled cultivation of *C. pasteurianum* grown on glycerol (D = 0.1 h^−1^ and ${c}_{s,in}$ = 36 g L^−1^)

ORP, mV	PDO, mmol mol^−1^	Eth, mmol mol^−1^	BuOH, mmol mol^−1^	Lac, mmol mol^−1^	Form, mmol mol^−1^	Ace, mmol mol^−1^	Buty, mmol mol^−1^	BM, mmol mol^−1^	C_R_, %	R_H_, %
−462	149 ± 4	26 ± 6	258 ± 5	0 ± 0	24 ± 1	13 ± 1	51 ± 1	1356 ± 322	89	85
−416	127 ± 4	31 ± 5	257 ± 10	28 ± 1	55 ± 2	18 ± 1	11 ± 1	981 ± 173	88	84
−337	183 ± 4	66 ± 20	183 ± 8	80 ± 2	66 ± 2	5 ± 11	23 ± 2	606 ± 196	84	80
−250	234 ± 2	129 ± 1	95 ± 0	209 ± 3	0 ± 0	0 ± 0	0 ± 0	239 ± 2	84^*^	81^*^

^*^CO_2_ and H_2_ production rates estimated from MFA

### Metabolic flux analysis and metabolomic data

While the product yields and final concentrations of the conducted experiments are important parameters to assess the overall process performance, these values can only hint at how the changed process conditions affected the cells' different pathways. Here, the data generated by MFA and the subsequent regulation analysis are more helpful. Also, by the mathematical representation of the assumed reaction network and the implementation of measured rates, MFA allows the statistical testing of consistency and the detection of gross measurement errors. The basis for the biochemical network used for MFA was developed within the framework of a previous publication [32]. ATP was not considered in the original network from the quoted work because it was expected to be directly consumed for growth. Since, based on previous results [29], it was of high interest to understand more about the energy metabolism of *C. pasteurianum*, ATP generation by substrate-level phosphorylation, and depletion by biomass formation were added to the network. The yield of biomass per mol ATP was taken from the literature [33]. Additionally, the whole model was charge and mass balanced. First, MFA results indicated that the measured rates and the initially assumed network of reactions are statistically inconsistent. This was concluded from the value of the test function *h*, which should yield values of < 7.815 (at a confidence interval of 95%) for the network, which has a *degree of redundancy* of three. No specific rate could be identified that contained gross systematic errors. Nonetheless, it could be concluded that intracellular protons cannot be sufficiently balanced by the assumed reactions at the measured rates. The main sink for protons in the network is the formation of H_2_ from reduced ferredoxin and protons. The primary source is the conversion of glycerol to pyruvate and acid production. In the following, possible reasons for the issue of proton deficiency are elucidated and treated in more detail.

In literature, it has been taken for granted that organic acids are mainly excreted passively in their membrane-permeable, protonated, and non-charged form, which is pH-dependent. This is also reflected in the transport reactions of most metabolic models. Nonetheless, this long-standing paradigm is currently under debate since it has been shown to contradict numerous experimental findings. In particular, it has been shown that most metabolic models underestimate the diversity of metabolite secretion and that simulation results do not agree with results of empirical exometabolomic studies [34]. Furthermore, in most of these studies, significant numbers of extracellularly quantified metabolites could not be explained by the metabolic model, even if leakage by cell lysis was considered as a source of error in exometabolomic studies [35]. This points out the future necessity to complement metabolic models for yet unknown transport reactions and mechanisms.

In this context, for *C. pasteurianum* the intracellular pH is expected to be more alkaline than the extracellular environment. Based on the data of Riebeling et al. [36], an intracellular pH of 6.5 is assumed at the applied external pH of 6.0. This leads to the situation that more than 98% of the main organic acids (butyric acids, acetic acid, lactic acid, formic acid) would be intracellularly present in their dissociated form. If these deprotonated acids could not be excreted by the cells, these metabolites would accumulate rapidly and severely inhibit cell growth, prevent steady state or even lead to a washout of cells in continuous cultures. Hence, it is expected that *C. pasteurianum* can excrete both forms of metabolites, protonated but also deprotonated. The metabolic model was adjusted accordingly: the degree of deprotonation was calculated with respect to the approximated intracellular pH of 6.5 and the pk_a_ values of the corresponding acid. Furthermore, each acid's protonated/deprotonated forms are only represented by one metabolite in the equations, which are both expected to be excreted by the cells. This enables the cells to balance significant parts of the proton deficiency created by the hydrogenases with the help of acid production. Nonetheless, the MFA results with the updated network, which includes the secretion of deprotonated acids, still did not deliver statistically satisfying results. The proton deficiency was still too significant.

**Fig. 3:**
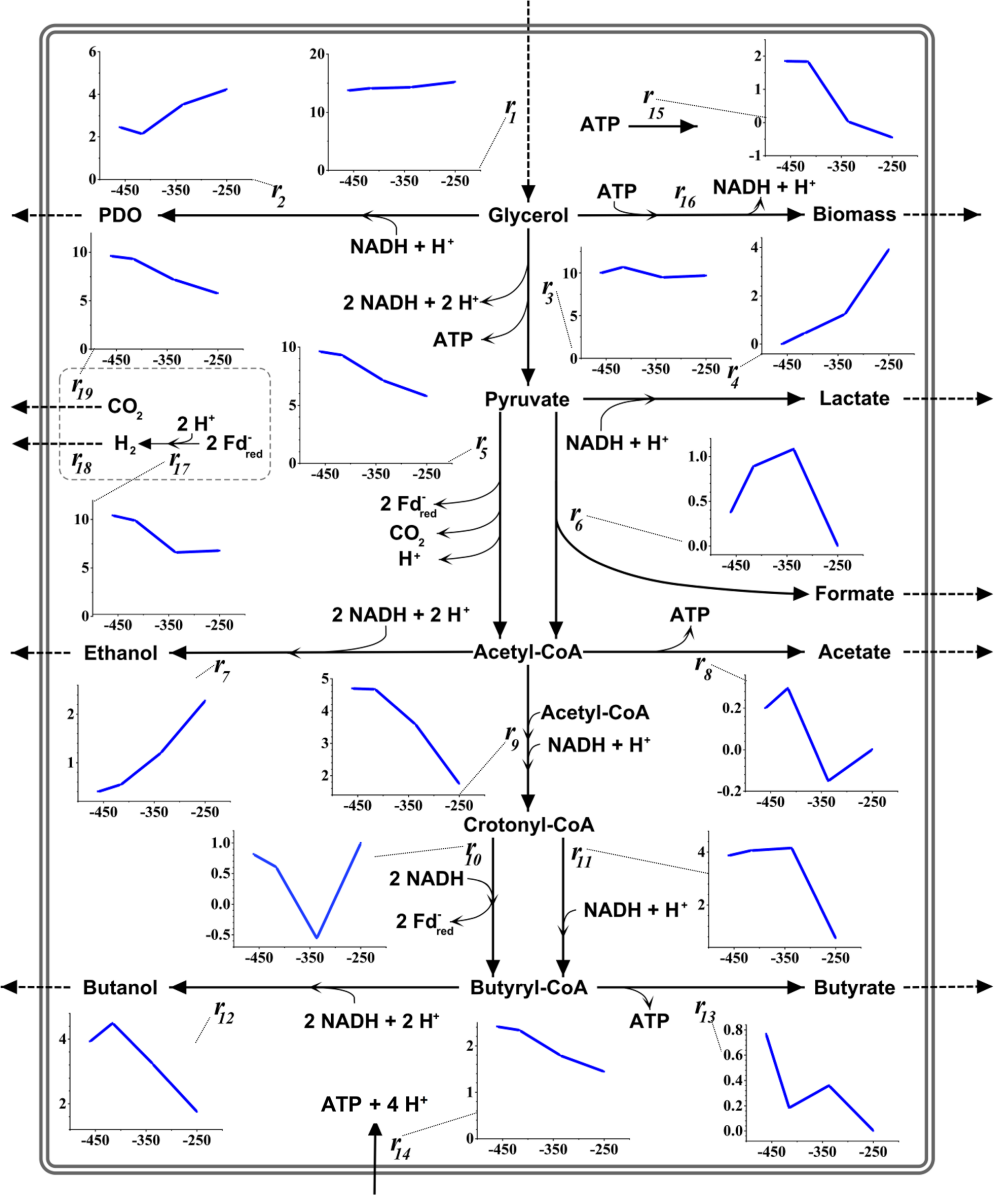
Results of the metabolic flux analysis during the continuous and ORP-controlled cultivation of *C. pasteurianum* in Biebl medium with glycerol (36 g L^−1^ feed concentration and D = 0.1 h^−1^). For better visualization, data points obtained from the flux analysis were connected by linear curves. For all graphs: y-axis gives the reaction rate in mmol g^−1^ h^−1^ and x-axis ORP in mV. For simplicity, only NADH, ATP, Ferredoxin (Fd_red_), and protons are shown as cofactors. Dashed lines indicate measured reaction rates at steady state. Solid lines indicate calculated rates

Subsequently, an ATPase was considered a further option for proton balancing by enabling proton flow into the cells. The F_1_F_0_ ATP synthase peptide of *C. pasteurianum* is well characterized [37], but only a few studies exist that elucidate its physiological role. It was concluded from these studies that the ATPase complex shows only low ATP-synthase activity and is inhibited by high ATP levels [38, 39]. Together with the finding that the intracellular pH of *C. pasteurianum* is more alkaline than its environment [36], this led to the assumption that the ATPase mainly uses ATP as an energetic driver to facilitate proton translocation out of the cells to maintain a more alkaline pH. Instead, it might also be an option that the hydrogenases establish the intracellular more alkaline pH, and the ATPase can then be used for ATP synthesis. Similar energy-conserving systems have been reported to exist in other microbes [40]. To examine this possibility for *C. pasteurianum*, a reaction equation for the ATPase was added to the metabolic model and MFA was conducted again. This time, when allowing proton influx and ATP generation by the ATPase, the statistical tests for data consistency with respect to the assumed metabolic model were satisfying for all tested conditions. The MFA results obtained by the final model are displayed in Fig. 3. All calculated values and the final model can also be found in an online data repository [41].

In this context, it must be pointed out that the intracellular pH, ATP demand for cell growth, and the amount of stoichiometrically produced ATP per proton (Y_ATP/H+_  = 0.25 mol mol^−1^) by the ATPase are only estimates from the literature. This leads to the situation that in one scenario at -250 mV, the reaction, which represents ATP requirements for maintenance (*r*_*15*_), slightly yielded a negative value in the calculations (artificial ATP generation). Hence, most likely, the actual ATP demand for growth is different from the value used in the model and varies among the tested scenarios. Moreover, it might also be that the ATPase efficiency was underestimated or that additional ATP-generating mechanisms existed in *C. pasteurianum*. Therefore, further experimental validation of the ATPase efficiency and improved metabolic models with more detailed biomass formulation equations are required in this relation. Nonetheless, the suggested mechanism of ATP generation, which is indirectly driven by the hydrogenase-established proton deficiency, explains how *C. pasteurianum* can generate sufficient ATP, even when only small amounts of acids are formed.

**Fig. 4:**
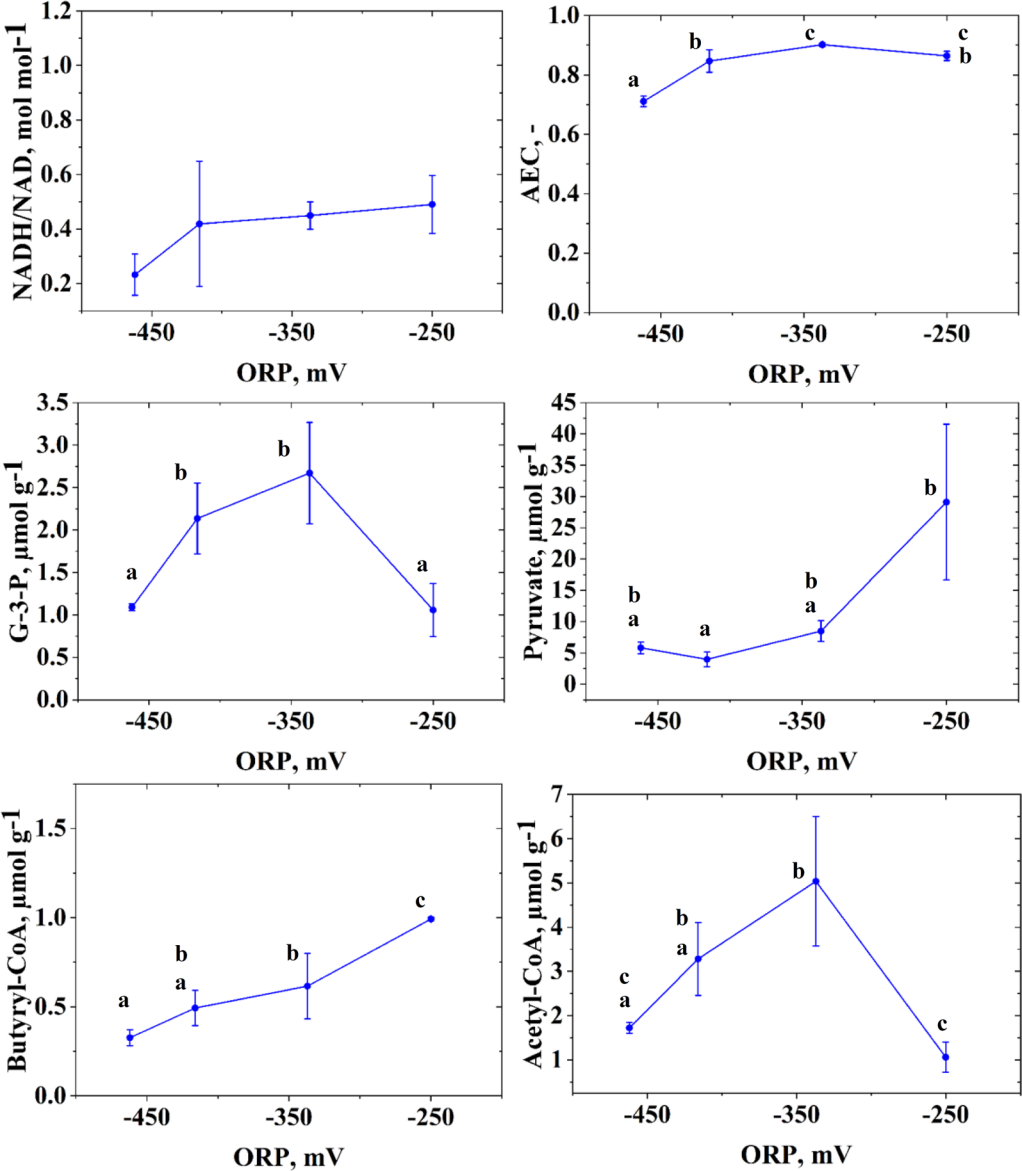
Molar NADH/NAD ratio, adenylate energy charge (AEC), and intracellular concentrations of glyceralaldehyde-3-phosphate (G-3-P), pyruvate, butyryl-CoA and acetyl-CoA during the continuous and ORP controlled cultivation of *C. pasteurianum* in Biebl medium with glycerol (36 g L^−1^ feed concentration and D = 0.1 h^−1^). Small letters above the blue curve denote that the data point belongs to a statistically significantly (α = 0.05) differing group. When no letters are shown in the subpanels, data did not differ significantly, as tested by ANOVA

The analysis of the metabolic data is shown in Fig. 4. The exact measures value for each sample is also made available in an online data repository [41]. Here, it is remarkable that the NADH/NAD ratio shows no statistically significant difference for the different tested ORP levels, demonstrating redox homeostasis. The AEC increases statistically significantly with a more positive ORP. This might result from growth inhibition by excess amounts of produced oxygen. The intracellular concentration of pyruvate increases strongly and significantly with higher ORP. Here, the cells showed a fivefold increase in pyruvate concentration by the transition from −337 mv to −250 mV. Since the concentration of acetyl-CoA decreased in the concerned ORP range, and also the reaction rate of the pyruvate-ferredoxin oxidoreductase (PFOR; *r*_*5*_ in Fig. 3) and pyruvate-formate lyase (PFL; *r*_*6*_ in Fig. 3) declined, the accumulation of pyruvate already hints towards an inhibition at the pyruvate node. The PFOR is well known to be inhibited by oxygen, presumably because the iron-sulphur cluster of the required cofactor ferredoxin is inactivated upon oxidation [42, 43]. But how exactly the ORP change influenced the intracellular reaction rates and how these changes were achieved (by mediated or direct effects) was evaluated more systematically and in more detail by the followingly presented sensitivity and regulation analysis.

### Sensitivity and regulation analyses

**Fig. 5:**
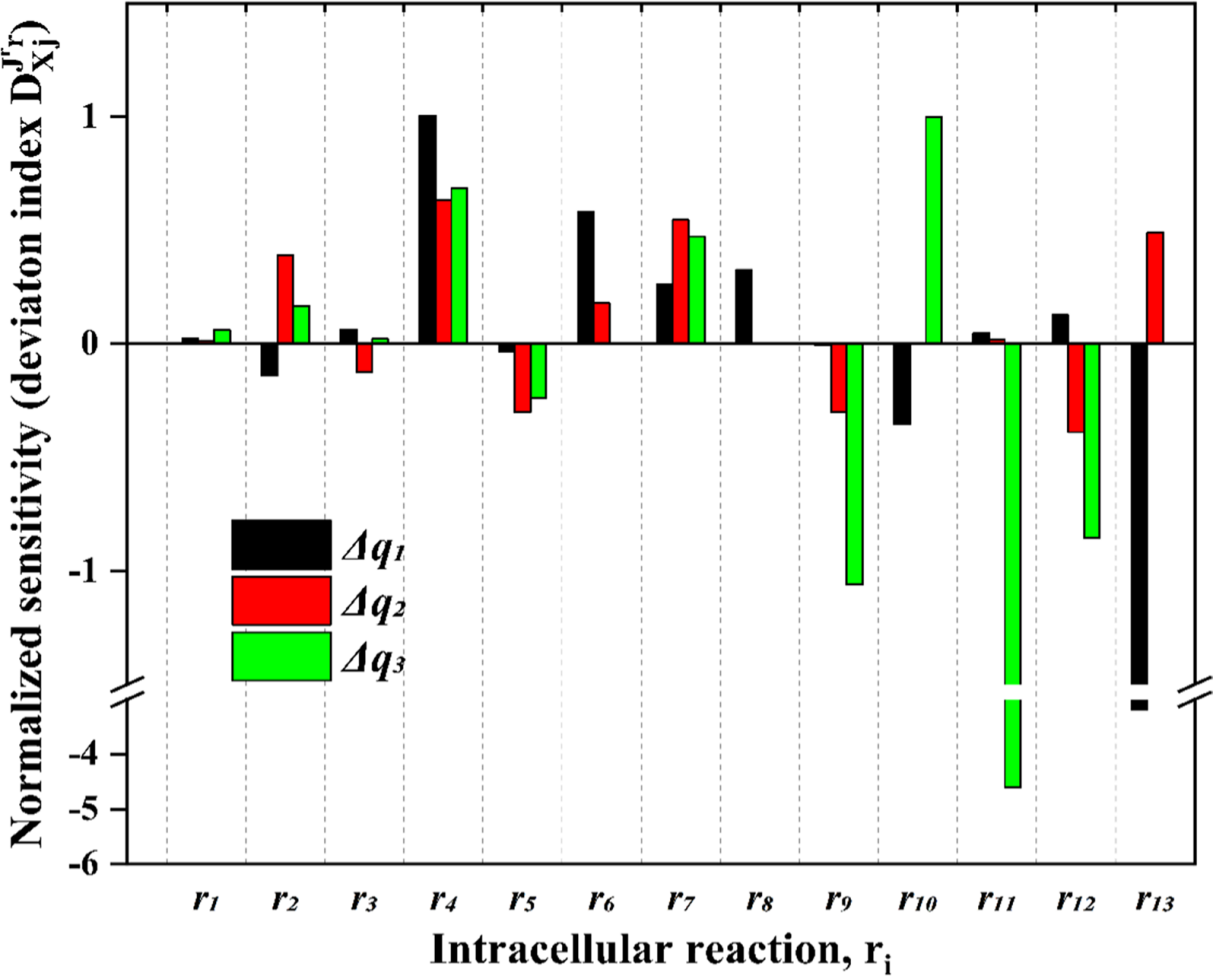
Sensitivity analysis of intracellular reactions in response to electrochemically-controlled ORP changes during the continuous cultivation of *C. pasteurianum* grown on glycerol. *Δq*_*1*_: -462 mV → -416 mV; *Δq*_*2*_: -416 mV → -337 mV; *Δq*_*3*_: -337 mV → -250 mV. If no value is shown, the reaction rate was zero at the new state and normalization is not possible

First, the deviation index was calculated for intracellular reactions *r*_*1*_ to *r*_*13*_ in response to the three ORP transitions. The results are shown in Fig. 5. Here, it is essential to notice that the values are normalized to the new steady. Therefore, the deviation indices for some rates with a very small absolute reaction rates – in particular *r*_*11*_ and *r*_*13*_ – display very extreme negative values. However, the sensitivity analysis shows that some reaction rates reacted to all transitions with a more positive ORP with the same trend: *r*_*4*_ (lactate formation) and *r*_*7*_ (ethanol formation) increased while *r*_*5*_ (PFOR) and *r*_*9*_ (crotonyl-CoA formation) declined. Reactions downstream of crotonyl-CoA (*r*_*10*_ to *r*_*13*_), formate and acetate formations (*r*_*6*_ and *r*_*8*_), and reactions upwards of pyruvate (*r*_*1*_ to *r*_*3*_) show a mixed picture. Interestingly, the substrate uptake rate (*r*_*1*_) appeared almost unaffected by the change in ORP levels.

**Fig. 6:**
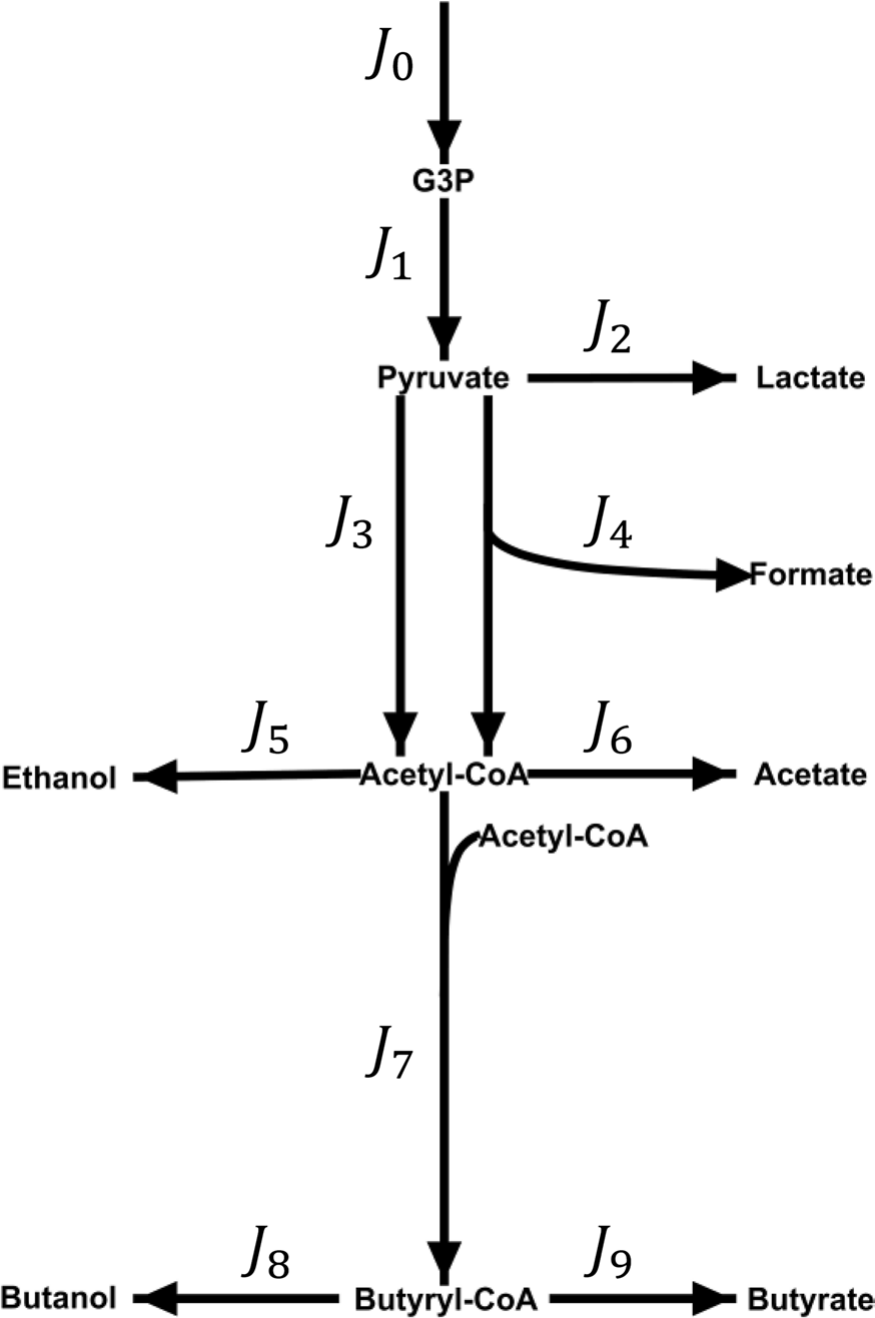
Reaction network used for symbolic MCA and regulation analysis. G3P = glyceralaldeyde-3-phosphate

**Fig. 7:**
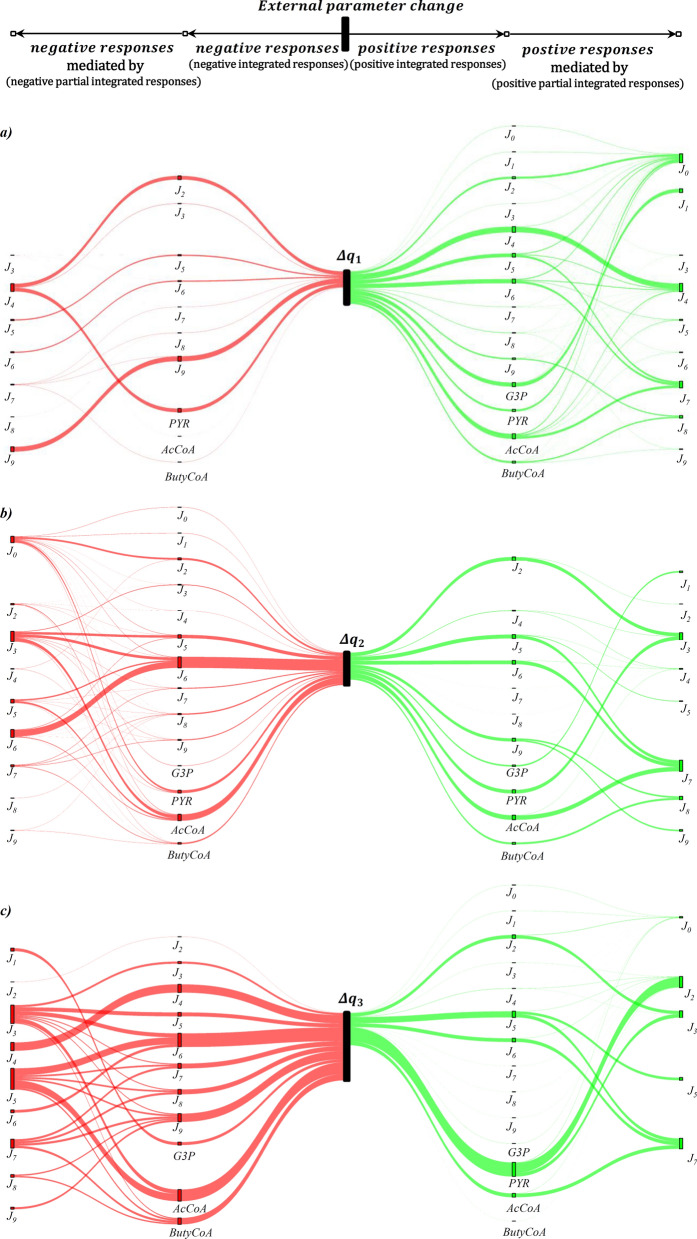
Visualization of regulation analysis in response to electrochemically-controlled ORP changes during the continuous cultivation (D = 0.1 h^−1^) of *C. pasteurianum* grown on glycerol. a) *Δq*_*1*_: − 462 mV → -− 16 mV; b) *Δq*_*2*_: − 416 mV → -− 37 mV; c) *Δq*_*3*_: − 337 mV → -− 250 mV. Green links indicate an activation/positive response of the flux or metabolite, and red links indicate an inhibition/negative response. Starting from the center node (*Δq*_*i*_), the first edges in both directions indicates the negative and positive response of a flux or intermediate (sum of negative and positive partial integrated responses). The second respective links towards the outer vertices shows through which flux the response was transmitted (equals partial integrated response). Thickness of the links correspond to the relative strength/value of the (partial) integrated responses. Missing of fluxes or intermediates in one scenario means that they were not affected by the external parameter change

The sensitivity analysis shows quantitatively which reaction rates are influenced by the ORP change. Still, it does not provide any information about the underlying control patterns and how the observed changes in reaction rates were achieved. Therefore, a regulation analysis was performed. For this, first, the system was simplified into blocks and redefined to cover only reactions for which intermediates were experimentally measured (G3P, pyruvate, acetyl-CoA and butyryl-CoA). The system is displayed in Fig. 6. For these reactions, the elasticities were estimated based on the intracellular concentration measurements and kinetic data from the literature (see Additional file 2: Table S2.1). Together with the obtained steady-state fluxes from the MFA, this enabled the calculation of concentration and flux control coefficients by symbolic MCA. The data of all four determined control patterns are supplemented (Additional file 2: Table S2.2). The regulation analysis results in the form of the (partial) integrated responses are listed in Table 3 and are visualized in Fig. 7. Each row in Table 3 lists the partial integrated responses and their sum (integrated response) in the last column. Hence, the rows indicate how much a system component (reaction block or intermediate) was positively or negatively affected by the respective ORP change. The columns show through which reaction block the observed change was achieved. For instance, for the first ORP change (Δq_1_) from − 462 mV to − 416 mV, reaction block *J*_*0*_ shows a slightly positive response, as indicated by the partial integrated response (${}I{R}_{{\Delta q}_{1}}^{{J}_{0}})$ of 0.07. Here, all partial integrated responses, except for reaction block *J*_*0*_ ($={}{}^{{J}_{0}}I{R}_{{\Delta q}_{1}}^{{J}_{0}}$), are zero. Hence, the observed effect was not transmitted through any reaction blocks other than *J*_*0*_ and is of direct nature. In contrast to this, *J*_*6*_ shows a positive response (${}I{R}_{{\Delta q}_{1}}^{{J}_{6}}=0.73$), but the partial integrated response for the reaction block itself (${}^{{J}_{6}}I{R}_{{\Delta q}_{1}}^{{J}_{6}}$) is negative. Therefore, the ORP negatively influenced the reaction block itself, but the observed overall positive response was transmitted through other reaction blocks. The strongest positive influence had block *J*_*7*_, which did not show an increased steady-state flux by the ORP change from − 462 mV to − 416 mV. Also, the positive response of *J*_*6*_ was transmitted through *J*_*0*_, *J*_*4*_ and *J*_*5*_. Figure 7 illustrates the described responses and also distinguishes between positive and negative responses. For ${}I{R}_{{\Delta q}_{1}}^{{J}_{0}}$ only one thin green line is drawn, which is only connected to *J*_*0*_ since there was only one slightly positive response that originated from *J*_*0*_ itself. For ${}I{R}_{{\Delta q}_{1}}^{{J}_{6}}$ the positive and negative parts of the integrated response are displayed and through which reaction blocks these were transmitted. Note that the thickness of the links in Fig. 7 refers to the absolute values of the (partial) integrated responses.

**Table 3:** Regulation analysis of intracellular reactions in response to electrochemically-controlled ORP changes during the continuous cultivation of *C. pasteurianum* grown on glycerol

${}^{i}I{R}_{\Delta q}^{a}$			i =										${}^{ }I{R}_{\Delta q}^{a}$
			0	1	2	3	4	5	6	7	8	9	
Δq_1_	a =	0	0.07	0.00	n.d	0.00	0.00	0.00	0.00	0.00	0.00	0.00	0.07
		1	0.07	0.00	n.d	0.00	0.00	0.00	0.00	0.00	0.00	0.00	0.07
		2	0.48	0.00	n.d	0.03	−0.87	0.00	0.00	0.00	0.00	0.00	−0.36
		3	0.04	0.00	n.d	0.00	−0.08	0.00	0.00	0.00	0.00	0.00	−0.04
		4	0.08	0.00	n.d	0.00	1.28	0.00	0.00	0.00	0.00	0.00	1.36
		5	0.20	0.00	n.d	−0.01	0.17	−0.29	0.04	0.47	0.00	0.00	0.58
		6	0.22	0.00	n.d	−0.01	0.18	0.10	−0.27	0.51	0.00	0.00	0.73
		7	0.03	0.00	n.d	0.00	0.03	0.01	0.01	−0.04	0.00	0.00	0.04
		8	0.03	0.00	n.d	0.00	0.03	0.01	0.01	−0.04	−0.01	0.05	0.07
		9	0.03	0.00	n.d	0.00	0.03	0.01	0.01	−0.04	0.27	−1.15	−0.84
		G3P	0.08	0.88	n.d	0.00	0.00	0.00	0.00	0.00	0.00	0.00	0.95
		PYR	0.49	0.00	n.d	0.03	−0.89	0.00	0.00	0.00	0.00	0.00	−0.37
		AcCoA	0.25	0.00	n.d	−0.01	0.21	0.11	0.05	0.58	0.00	0.00	1.19
		ButyCoA	0.04	0.00	n.d	0.00	0.03	0.02	0.01	−0.05	0.32	0.06	0.42
Δq_2_	a =	0	−0.11	0.00	0.00	0.00	0.00	0.00	0.00	0.00	0.00	0.00	−0.11
		1	−0.11	0.00	0.00	0.00	0.00	0.00	0.00	0.00	0.00	0.00	−0.11
		2	−0.65	0.00	0.08	1.27	−0.08	0.00	0.00	0.00	0.00	0.00	0.62
		3	−0.03	0.00	−0.01	−0.23	0.00	0.00	0.00	0.00	0.00	0.00	−0.27
		4	−0.05	0.00	−0.02	0.09	0.11	0.00	0.00	0.00	0.00	0.00	0.13
		5	−0.13	0.00	−0.05	−0.85	0.05	0.23	−0.17	1.13	0.00	0.00	0.20
		6	−0.15	0.00	−0.06	−0.97	0.06	−0.50	−2.32	1.29	0.00	0.00	−2.65
		7	−0.02	0.00	−0.01	−0.10	0.01	−0.05	−0.02	−0.16	0.00	0.00	−0.34
		8	−0.01	0.00	−0.01	−0.10	0.01	−0.05	−0.02	−0.16	−0.06	−0.06	−0.47
		9	−0.02	0.00	−0.01	−0.10	0.01	−0.05	−0.02	−0.17	0.57	0.55	0.77
		G3P	−0.14	0.56	0.00	0.00	0.00	0.00	0.00	0.00	0.00	0.00	0.43
		PYR	−0.68	0.00	−0.26	1.32	−0.08	0.00	0.00	0.00	0.00	0.00	0.30
		AcCoA	−0.19	0.00	−0.07	−1.20	0.07	−0.61	−0.25	1.59	0.00	0.00	−0.66
		ButyCoA	−0.02	0.00	−0.01	−0.12	0.01	−0.06	−0.03	−0.21	0.71	−0.08	0.19
Δq_3_	a =	0	0.02	0.00	0.00	0.00	0.00	0.00	0.00	0.00	0.00	0.00	0.02
		1	0.02	0.00	0.00	0.00	0.00	0.00	0.00	0.00	0.00	0.00	0.02
		2	0.05	0.00	−0.02	0.48	0.00	0.00	0.00	0.00	0.00	0.00	0.51
		3	0.00	0.00	0.02	−0.33	0.00	0.00	0.00	0.00	0.00	0.00	−0.31
		4	0.00	0.00	0.03	0.01	−1.26	0.00	0.00	0.00	0.00	0.00	−1.22
		5	0.00	0.00	0.03	−0.59	0.00	0.40	0.00	0.51	0.00	0.00	0.34
		6	0.00	0.00	0.03	−0.62	0.00	−1.06	−0.42	0.53	0.00	0.00	−1.54
		7	0.00	0.00	0.01	−0.15	0.00	−0.26	0.00	−0.33	0.00	0.00	−0.73
		8	0.00	0.00	0.01	−0.15	0.00	−0.26	0.00	−0.33	0.00	0.00	−0.73
		9	0.00	0.00	0.01	−0.15	0.00	−0.26	0.00	−0.33	−0.20	−0.31	−1.25
		G3P	0.02	−0.45	0.00	0.00	0.00	0.00	0.00	0.00	0.00	0.00	−0.43
		PYR	0.06	0.00	1.51	0.57	0.00	0.00	0.00	0.00	0.00	0.00	2.14
		AcCoA	0.00	0.00	0.03	−0.66	0.00	−1.12	0.00	0.56	0.00	0.00	−1.19
		ButyCoA	0.00	0.00	0.01	−0.16	0.00	−0.27	0.00	−0.34	−0.21	0.00	−0.97

*Δq*_*1*_: − 462 mV → − 6 mV; b) *Δq*_*2*_: − 416 mV → -− 337 mV; c) *Δq*_*3*_: − 337 mV → -− 250 mV. $${}^{i}I{R}_{\Delta q}^{a}$$= integrated partial response; $${}I{R}_{\Delta q}^{a}=\sum {}{}^{i}I{R}_{\Delta q}^{a}$$ = integrated response; G3P = glyceralaldehyde-3-phosphate; PYR = pyruvate; AcCoA = acetyl-CoA; ButyCoA = butyryl-CoA; n.d. = not defined (since $${J}_{i}^{0}=0)$$. a stands for a system variable (flux and metabolite level), which response to the parameter change through block i. For further explanation, see text

The data enable interesting yet highly complex insights into the response of the microbial system to the ORP changes. For the first transition (from −462 mV to −416 mV, a) in Fig. 7), the positive response of the system outweighs the adverse effects. Here, the most significant overall positive effects, as reflected by the integrated responses, are seen for *J*_*4*_, *J*_*6*_, G3P and acetyl-CoA. While the activation of *J*_*4*_ is mainly of direct nature, *J*_*6*_ profits, as already described, most from the increase in *J*_*0*_ (system influx) and *J*_*7*_ (conversion of acetyl-CoA to butyryl-CoA), which remained almost unchanged by the parameter change from −462 mV to −416 mV. Also, the increase in the rate of the PFL (*J*_*4*_) has a positive effect on *J*_*6*_. G3P concentration does almost exclusively profit from the slightly increased system influx. The concentration of acetyl-CoA is positively affected by reaction block *J*_*7*_, which did not show an increased flux compared to the reference state. Accordingly, fluxes *J*_*5*_ and *J*_*6*_ did not increase sufficiently to compensate for the local increase in acetyl-CoA concentration caused by the rise in *J*_*4*_. For the first ORP change from −462 mV to −416 mV, the highest negative responses are seen for *J*_*2*_, *J*_*9*_ and pyruvate. While the negative effect on lactate production (*J*_*2*_) can entirely be explained by the increase of flux for PFL (*J*_*4*_*)*, the negative response of *J*_*9*_ appears to be of direct nature. The negative effect of the effector change on pyruvate can also mainly be explained by an increase in *J*_*4*_.

The second change in external effector (transition from −416 mV to −337 mV; b) in Fig. 7) has an overall negative influence on the system. Here, *J*_*6*_ show the highest negative response. The response is transmitted directly but also through *J*_*3*_ (PFOR), which is reduced, and *J*_*5*_ (ethanol formation)*,* which is increased. In this context, it is noteworthy that the decrease in the flux of *J*_*3*_ that exerts a strong influence on several system components does almost exclusive occur from direct response. Only a small proportion (11%) can be explained by a reduced system influx after the transition. The highest overall positive response (as indicated by the total integrated response ${}I{R}_{\Delta q}^{a}$) can be recognized in *J*_*2*_ and *J*_*9*_. Here, *J*_*2*_ (lactate formation) increases mainly because of the negative response of *J*_*3*_ (PFOR) and *J*_*9*_ is activated directly and positively affected by less butanol production (*J*_*8*_). The observed increase in pyruvate and G3P concentrations (as shown in Fig. 4) can mainly be attributed to the decrease of *J*_*3*_ and *J*_*1*_, while acetyl-CoA is mostly negatively affected by a reduction in *J*_*3*_.

For the third ORP change (from −337 mV to −250 mV, c) in Fig. 6), the negative response of the system clearly predominates. For the fluxes, only *J*_*2*_ (lactate formation) and *J*_*5*_ (ethanol formation) show an overall positive response. This time, the positive response of *J*_*2*_ is again mainly transmitted by a further decrease in *J*_*3*_. *J*_*5*_ profits from a direct activation and also from a substantial flux reduction in reaction block *J*_*7*_*.* The highest negative responses were calculated for *J*_*4*_ (PFL), *J*_*6*_ (acetate formation) and *J*_*9*_ (butyrate formation). PFL is, identical to PFOR during this final ORP change, solely affected directly by the change of the external effector. In general, PFOR is engaged in transmitting the negative effects of the external effector on all reactions and intermediates downstream of it. Also, the decreasing value of *J*_*3*_ is the main reason for the observed concentration increase of pyruvate and the increase in lactate formation (*J*_*2*_).

Overall, in combination with the MFA results, the regulation analysis draws a cohesive picture of the metabolic response to the ORP alterations. Although the applied method is hampered by the derivation of the elasticities from kinetic data from literature and the assumption of a simple one substrate Michaelis–Menten kinetic, it still enables a reliable semi-quantitative elucidation of the triggered metabolic phenomena and how they were achieved from a systems level perspective. As represented by the analysis of the first change in ORP parameter (−462 mV to −416 mV), a moderate increase of ORP stimulates the metabolism of *C. pasteurianum* and results in an increased substrate influx towards pyruvate. Here, the reaction of PFOR remains almost unaltered and the PFL captures the additional flux towards pyruvate. To maintain redox homeostasis, the cells produce more ethanol. The following ORP change (from −416 mV to −337 mV) results in a drastic inhibition of PFOR, presumably caused by a higher oxygen activity, driven by the higher amounts of applied currents. Here, the cells again utilize the PFL reaction to compensate the lower PFOR activity. In addition, upstream of pyruvate more lactate is produced as a response. At the highest ORP level (−250 mV) and the highest activity of oxygen in the system, not only PFOR but also PFL are directly inhibited. Both enzymes carry iron sulphur clusters and are inhibited by molecular oxygen [44, 45]. However, it is interesting to notice that PFL inhibition is first observed at the highest effector level, while PFOR is negatively affected already at lower levels. In response to the strong blockage of pyruvate conversion to acetyl-CoA, the cells shuffle even more molecules towards lactate. Although ethanol yields still increase for the last ORP shift, the regulation analysis indicates that the decrease in the PFOR reaction rate exerts a strong negative effect on ethanol formation. Moreover, even though lactate yields are more than doubled in the last ORP parameter change, the cells are obviously unable to re-oxidize the required amounts of NADH, which are produced during glycerol conversion and biomass formation. Hence, the direct inhibition of pyruvate conversion to acetyl-CoA (PFOR and PFL) by the external effector oxygen indirectly causes the observed increase in PDO yield. The level of this effector in the system can be controlled by the developed algorithm for electrochemical ORP control.

## Conclusions

In this work, we successfully developed a system for electrochemical anodic ORP control during the continuous cultivation of *C. pasteurianum*. An increase in PDO yield of 57% was observed by a stepwise increase of ORP from −462 mv to −250 mV at a dilution rate of 0.1 h^−1^. The ORP increase was driven by the electrochemical production of oxygen, which on the one hand, directly negatively influenced the conversion of pyruvate to acetyl-CoA, and on the other hand, positively influenced product yields of compounds upstream of pyruvate. Therefore, the developed technique might also be of interest to other anaerobic or microaerobic processes. However, since the artificial ORP increase also resulted in lower biomass and product concentrations, it is essential for future application of this approach to find the right balance between product yield and volumetric productivity. Furthermore, to optimize the process specifically for *C. pasteurianum*, more detailed data about the *in vivo* inhibition kinetics of PFOR and PFL are required. Also, it would be beneficial to get improved data on the related control patterns by not deriving the elasticities from kinetic data from literature and the metabolite concentrations but directly experimentally. This could be achieved by experiments in which parallel short-term perturbations (similar to Tröndle et al. [46]) are applied, or enzyme concentrations are determined at steady state, which would allow the calculation of the coefficients by the lin-log method [47]. Nonetheless, the first approach (short-term perturbation experiments) would require further improvement of the electrochemical ORP-control under more dynamic conditions.

## Materials and methods

### Microorganism and medium

An electrocompetent mutant of the wild-type strain *C. pasteurianum* DSM 525 was used in this work. The strain was generated by Schmitz et al. [32] and is named *C. pasteurianum* R525. Two media were used for pre-culture growth and fermentation: Reinforced Clostridial Medium (RCM) and a modified medium from Biebl [48]. Strain maintenance and media preparation for the pre-cultures followed the same procedures as described by Sabra et al. [49]. First, 1 mL of culture from cryovials was grown for 24 h in RCM medium, and then 1.5 mL were transferred into a second pre-culture anaerobic bottle containing 50 mL Biebl medium with 5 g L^−1^ yeast extract. The second pre-culture was then used for inoculation of the main culture. The main culture medium and also the feeding solution contained Biebl medium, but yeast extract was replaced with 25 µg L^−1^ biotin. The initial concentration of glycerol in the main culture was 25 g L^−1^. The feed medium contained 36 g L^−1^ of glycerol. FeSO_4_·7 H_2_O, biotin and L-cysteine were prepared separately and added by sterile filtration into the main culture medium and the feeding solution. The feed medium was autoclaved in 10 L tanks and purged with nitrogen while passively cooling down to room temperature to establish anaerobic conditions. Anaerobic conditions were verified by the addition of the dye resazurin. The fermentation and feed medium also contained 1 g L^−1^ of D-xylose as an internal standard for calculating the cell-specific concentration of intracellular compounds obtained from automated fast-filtration. Cultures and feeding solutions were regularly examined under a light microscope to check for contamination.

### Cultivation systems and conditions

The fermentations for the continuous electricity-aided cultivations were conducted in a glass bioreactor from Bioengineering coupled to a self-developed fast-filtration system [50]. Here, the reactor had a working volume of 1.4 L. This volume was the minimal volume possible to ensure that the platinized-titan mesh electrode of the used "All-in-One" electrode was always covered with liquid. Preparations (probe calibration, sterilization, and establishing anaerobic conditions) and process conditions (pH, temperature, stirring rate) were the same as described in previous work [29]. Off-gas composition (H_2_, CO_2_, N_2_, O_2_) and volume stream were measured by a Balzer Omnistart GSD 300 mass spectrometer coupled to highly accurate EL-FLOW mass flow meters from Bronkhorst (Netherlands). Feeding was started after an initial batch phase of 17 h. It was assumed that steady-state conditions were achieved after a minimum of five hydraulic retention times, which was verified by an unchanging off-gas signal. Then, four samples for the quantification of extracellular metabolite concentrations and biomass were taken in 15-min intervals for each tested condition. After the fourth sampling, fast-filtration for gaining of the metabolomic data was conducted. Different ORP set-points were tested in the continuous cultivation. The control algorithms for ORP control by applying electric current had to be developed and further optimized during the experiments. Hence, the ORP control is considered part of the results section. The OPR control algorithm was implemented by a PID-function via the software Labview from National Instruments (US). The control algorithm used the online ORP value as input parameters to control a Gamry Interface 1000 potentiostat. Applied current and duration of electric pulses were defined as output parameters.

### Determination of cell growth and cell dry weight

Cell growth was tracked by measuring the OD_600_. Cell dry weight (*c*_*x*_) was determined gravimetrically in triplicates from cell pellets of 2 mL culture broth, dried at 70 °C for 24 h. The pellets were obtained by 5 min of centrifugation at 13,000 rpm.

### Quantification of extracellular compounds

Glycerol, 1,3-propanediol (PDO), lactate, formate, acetate, ethanol, butyrate, pyruvate, succinate, and n-butanol were quantified by an HPLC system from Kontron (UK). Separation took place on Aminex HPX-87H column at 60 °C with a flow rate of 0.6 mL min^−1^ with 5 mM H_2_SO_4_ as the mobile phase. For this purpose, cells were centrifuged at 13,000 rpm for 5 min and the supernatant was filtered through a 0.2 µm PVDF filter. The ethanol concentrations were additionally verified using a commercially available enzymatic "Ethanol-Kit K-ETOH" from the company Megazyme (Ireland).

### Targeted metabolomics

For metabolomic investigations, an automated-fast filtration system was used, which was described in detail by da Luz et al. [51]. The exact sampling procedure, extraction procedure, and analytical method were the same as in our previous work [29]. In brief, the extraction method and protocol were taken and modified from Lu et al. [52], filters were quenched with liquid nitrogen and freeze-dried after two rounds of extraction. The samples were then resuspended in ultra-pure water and used for subsequent absolute LC-MS/MS and enzymatic quantification. For each steady-state condition, four filtrations were conducted and extracted separately. Each sample was measured in duplicate. The concentrations of glyceraldehyde-3-phosphate (G3P), NAD, NADH, ATP, ADP, AMP, pyruvate, acetyl-CoA and butyryl-CoA were determined.

### Calculations and statistical tests

Concentration data obtained from the extracellular steady-state measurements of substrate (*c*_*s*_) and products (*c*_*p*_) were used to calculate the cell-specific rates for substrate uptake (*q*_*s*_) and product formation (*q*_*p*_), according to the following equations:

1 $${q}_{S} =\frac{ D \left({c}_{s,in}-{c}_{s}\right) }{{c}_{x}}$$

2 $${q}_{p}= \frac{D \cdot  {c}_{P}}{{c}_{x}}$$

where D is the dilution rate in h^−1^ and ${c}_{s,in}$ the substrate concentration of the inlet flow in g L^−1^. The cell-specific rates were then used for the calculation of carbon and reducing equivalent balances. Carbon (*C*_*R*_) and macroscopic electron (*NADH*_*R*_) recoveries were obtained from the following equations [30, 53]:

3 $${C}_{R}=\,({3 q}_{PDO}+{2 q}_{Ethanol}+{4 q}_{Butanol}+4 {q}_{Butyrate}+ {2 q}_{Acetate}+ {q}_{Formate}+ 3 {q}_{Lactate}+ 4 {q}_{BM}+ {q}_{{CO}_{2}})\cdot \frac{1}{3 {q}_{Glycerol}}$$

4 $${R}_{H}^{MACRO}= \,{(16 q}_{PDO}+{12 q}_{Ethanol}+{24 q}_{Butanol}+20 {q}_{Butyrate}+ {8 q}_{Acetate}+ 2{ q}_{Formate}+ 12 {q}_{Lactate}+ 16 {q}_{BM}+ 2 {q}_{H2})\cdot \frac{1}{14 {q}_{Glycerol}}$$

The off-gas rates for CO_2_ (*q*_*CO2*_) were corrected for absorption in the liquid phase, according to Zeng [54]. The amount of measured H_2_ in the off-gas stream was also corrected for electrochemically produced gasses. The Adenylate Energy Charge (*AEC*) was obtained from the measured intracellular molar concentrations of AMP (*c*_*AMP*_), ADP (*c*_*ADP*_) and ATP (*c*_*ATP*_) by the following equation [55]:

5 $$AEC=\frac{{c}_{ATP}+ {0.5 c}_{ADP}}{{c}_{ATP}+{c}_{ADP}+{c}_{AMP}}$$

One-way analysis of variance (ANOVA) was conducted for testing the statistical significance of the obtained metabolomic data, followed by a posthoc Tukey's test. The analysis was performed with the Software Origin Version 2020 (OriginLab Corp., USA). Four data points were available for each metabolite from one steady-state condition. The significance level (α) was chosen to be 5%.

### Metabolic flux analysis

For convenience and simple modification plus testing of different biochemical networks, MFA was conducted with the Matlab plugin *CellNetAnalyzer* (Version 2017.3; [56]). The final biochemical network is described in Additional file 1 and the MFA results are available in an online data repository [41]. For rate estimation by *variances-weighted least square regression*, the variances of four independent measurements (taken in three 15 min intervals) at steady state were used for each condition. For off-gas values and the rate for biomass formation reaction, a standard deviation of 5% was assumed. The final metabolic network used for MFA has a *degree of redundancy* of three. This allowed for testing the measured rates and scenarios for consistency. In one case (−250 mV) the gas production rate was too low (< 5 mL min^−1^) to be measured by the off-gas MS. Here, the *degree of redundancy* was only one and the cell-specific production rates for H_2_ and CO_2_, required for the calculation of C_R_ and R_H_, were estimated by MFA.

### Sensitivity analysis

To quantitatively describe the response of metabolic systems to large (finite) changes in external factors, Small and Kacser [57] introduced the concept of deviation indices. In this context, a deviation index for the response of a reaction to a large (finite) change in an external factor can be described by:

6 $${D}_{{x}_{J}^{r}}^{{J}^{r}}=\left(\frac{\Delta J}{\Delta {X}_{J}}\right)\cdot  \frac{{X}_{J}^{r}}{{J}^{r}}$$

where ${D}_{{x}_{J}^{r}}^{{J}^{r}}$ is the deviation index of flux $J$ to a large change in the concentration of an external effector $X$. Furthermore, the following relations apply:

7 $$\Delta J= {J}^{r}-{J}^{0}$$

8 $$\Delta {X}_{J}= {X}_{J}^{r}-{X}^{0}$$

where $${J}^{0}$$ and $${X}^{0}$$ are flux and concentration at a reference state and $${J}^{r}$$ and $${X}_{J}^{r}$$ the corresponding values after an $$r$$-fold change of the external effector ($${X}_{J}^{r}=r \cdot  {X}^{0}$$). In fact, Eq. 6 equals the sensitivity of flux to an external effector, normalized to flux and concentration at the new state. To quantify the effect of ORP change on the intracellular fluxes, the question arises which species and values should be chosen for the external effector $$X$$. Since it was expected that oxygen, produced by the used platinized-titan electrodes, was the main contributor to the increase in ORP value, the activity of oxygen ($${a}_{{O}_{2}})$$ in the broth could be calculated for different redox levels by the following equation [3]:

9 $${a}_{{O}_{2}}={e}^{\left(\frac{\left({E}_{h}-{E}^{0}\right) \cdot  F}{R \cdot  T} + 2.303 \cdot  pH\right) \cdot  \,4}$$

where ${E}_{h}$ is the measured ORP value in V,${E}^{0}$ is the standards reduction potential of the O_2_/H_2_O couple (1.026 V vs. Ag/AgCl), F the Faraday constant, R the ideal gas constant, T the temperature and $pH= -\mathrm{log}({c}_{{H}^{+}})$. For the sensitivity analysis, ${a}_{{O}_{2}}$ was determined for all tested ORP values, used as the value for the external effector $X$, and ${D}_{{x}_{J}^{r}}^{{J}^{r}}$ calculated for all intracellular fluxes, which were previously obtained from the MFA. However, since the ORP was increased stepwise in four steps from − 462 mV to − 250 mV (− 462 mV →  − 416 mV → − 37 mV →  − 250 mV), the reference state for the calculations was changed to each new steady state, respectively. Hence, the results do not all reflect the response to the reference state without electricity (− 462 mV) but to the ORP value of the previous steady state.

### Regulation analysis

In relation to MCA, a regulation analysis provides a quantitative description of how a parameter causes changes in the fluxes and metabolite levels of metabolic systems and how they transmit through the system [58]. To perform a regulation analysis, steady-state fluxes, elasticities and control coefficients of the defined system must be known. In MCA, the elasticity of a metabolic process $i$ to a metabolite $x$ is given by:

10 $${\varepsilon }_{x}^{i}= \frac{d{v}_{i}}{dx}\cdot \frac{x}{{v}_{i}}$$

where ${v}_{i}$ denotes the rate of reaction of the metabolic process $i$. Hence, the elasticities reflect the local kinetics in relation to the metabolite level. Here, the metabolic process can be a single reaction or a series of lumped reactions. The control coefficient is defined as:

11 $${C}_{i}^{a}= \frac{da}{d{v}_{i}}\cdot \frac{{v}_{i}}{a}$$

where $a$ can be a system flux or a metabolite concentration. When $a$ is a flux, the coefficient is termed a flux control coefficient, and when $a$ is a metabolite concentration, it is called a concentration control coefficient.

Symbolic MCA represents an extension to the classical metabolic control analysis and enables the analytical calculation of algebraic control coefficient expressions [59]. Based on several known summation and connectivity relationships between control coefficients and elasticities, for small and simple linear systems, these expressions can be derived manually by hand. For larger and more complex systems, the algebraic expression might cover hundreds of terms and can be computed with the help of appropriate software. In this work, symbolic MCA was performed with the tool SymCA within the PySCeSToolbox [60, 61]. The Jupyter Notebook with the symbolic MCA code and the simplified MCA modelre available via an online data repository [41]. For calculation of the control coefficients, steady-state flux data from the MFA were used, and the elasticities were estimated from the measured intracellular metabolite concentrations and kinetic data from the literature (for details see Additional file 2). After this, as a first step, the integrated elasticities for each block ($I{E}_{\Delta q}^{i}$) were calculated by [62]:

12 $$I{E}_{\Delta q}^{i}= \Delta {J}_{i}-{\varepsilon }_{x}^{i}\cdot  \Delta x$$

with the elements:

13 $$\Delta x= \frac{{x}^{\Delta q}-{x}^{0}}{{x}^{0}}$$

14 $$\Delta {J}_{i}= \frac{{J}_{i}^{\Delta q}-{J}_{i}^{0}}{{J}_{i}^{0}}$$

where $x$ and $J$ denote the metabolite concentration and fluxes at perturbed (${x}^{\Delta q}, { J}_{i}^{\Delta q}$) and reference steady state (${x}^{0}, {J}_{i}^{0}$). The integrated elasticities quantify the amount in observed flux that cannot be explained locally by the change of the intermediates. They are related to the partial integrated responses ($^{i} IR_{\Delta q}^{a}$) by the beforehand calculated control coefficients [62]:

15 $$^{i} IR_{\Delta q}^{a} = C_{i}^{a} \cdot IE_{\Delta q}^{i} $$

These partial integrated responses state, to which extend the process $i$ is involved in transmitting the response in the variable $a$ (which is a flux or intermediate) to a parameter change ($\Delta q$). Therefore, they allow a conclusion if the observed change is of direct or indirect (transmitted) nature. Overall, the sum of the partial integrated responses quantifies the total strength of the observed response of the variable $a$ to the parameter change $\Delta q$. This sum is called the integrated response ($IR_{\Delta q}^{a}$):

16 $$ IR_{\Delta q}^{a} = \mathop \sum \limits_{all i} {}_{ }^{i} IR_{\Delta q}^{a} $$

## Supplementary Information

**Additional file 1: Table S1.1. **Metabolic model used for *metabolic flux analysis *for the continuous and ORP controlled fermentations of *Clostridium pasteurianum *.

**Additional file 2: Table S2.1. **Estimation of elasticities from kinetic literature data and measured metabolites in *C. pasteurianum*. **Table S2.2. **Calculated flux and concentration control coefficients of *C. pasteurianum*.

## Data Availability

The dataset supporting the conclusions of this article is available in the following Mendeley Data repository: https://data.mendeley.com/datasets/7cwf6mf33z/draft?a=34b0fe00-45fc-421a-bb74-fa37b968a071.
