# Supplementary material for: Control of redox potential in a novel continuous bioelectrochemical system led to remarkable metabolic and energetic responses of Clostridium pasteurianum grown on glycerol
Source: Microb Cell Fact. 2022 Sep 1;21:178. doi: 10.1186/s12934-022-01902-5 (PMC9434860; doi:10.1186/s12934-022-01902-5)
Supplement: Supplementary file 1 — Additional file 1: Table S1.1. Metabolic model used for metabolic flux analysis for the continuous and ORP controlled fermentations of Clostridium pasteurianum . [file 12934_2022_1902_MOESM1_ESM.pdf]

Supplementary S1

Control of redox potential in a novel continuous bioelectrochemical system led to remarkable metabolic and energetic responses of *Clostridium pasteurianum* grown on glycerol: Metabolic model

Philipp Arbter, Niklas Widderich, Tyll Utesch, Yaeseong Hong and An-Ping Zeng*

Institute of Bioprocess and Biosystems Engineering, Hamburg University of Technology, Denickestraße 15, D-21073 Hamburg, Germany

*corresponding author

Explanations and comments on the metabolic model (Table S1.1):

- The protonation of all acids at the assumed intracellular pH of 6.5 was calculated according to their pk_a values. Those are: 2.49 for pyruvate, 3.77 for formate, 4.76 for acetate, and 4.82 for butyrate. It was assumed that acids could be exported in their protonated but also deprotonated form (as described in the main text). This results in the occurrence of additional protons in the reaction equations of r_4, r_6, r_8, and r_{13}.
- When pyruvate is expected to be utterly deprotonated in the cells, the *pyruvate-ferredoxin oxidoreductase* (r_5) produces one proton (compare Lin et al. (2015), McAnulty et al. (2012) and Yoo et al. (2015)).
- In the bifurcating reaction of crotonyl-CoA to butyryl-CoA, no protons are required, when NADH is utilized (Buckel and Thauer, 2018; Lin et al., 2015).
- For the biomass equation, it was assumed that 1 mol Biomass equals 1 mol $C_4H_7O_2N$. The ATP demand of 11.67 mol mol^{-1} was taken from literature from *C. acetobutylicum* (compare doi: 10.1021/bk-1997-0666.ch014 (CHEN et al., 2006; Zeng et al., 1997).
- A value of 0.25 mol ATP per mol H^+ was used for the ATPase reaction (r_{14}). This value was estimated from literature from *C. authotheganum* (Müller et al., 2018; Valgepea et al., 2017).
- Reaction r_{15} accounts for energy requirements for maintenance.

Table S1.1: Metabolic model used for *metabolic flux analysis* for the continuous and ORP controlled fermentations of *Clostridium pasteurianum* (additional explanations are provided on the previous page). (ex) denotes extracellular compounds, all others are cytosolic.

r_i		Educts		Products
r_1	:	Glycerol (ex)	$\longrightarrow$	Glycerol
r_2	:	Glycerol + NADH + H^+	$\longrightarrow$	1,3-Propanediol + NAD^+ + H_2O
r_3	:	Glycerol + ADP + P_i + 2 NAD^+	$\longrightarrow$	Pyruvate + ATP + 2 NADH + 2 H^+ + H_2O
r_4	:	Pyruvate + NADH + 1.003 H^+	$\longrightarrow$	Lactate + NAD^+
r_5	:	Pyruvate + CoA + Fd	$\longrightarrow$	Acetyl-CoA + Fd^{2-} + CO_2 + H^+
r_6	:	Pyruvate + CoA + 0.002 H^+	$\longrightarrow$	Acetyl-CoA + Formate
r_7	:	Acetyl-CoA + 2 NADH + 2 H^+	$\longrightarrow$	Ethanol + CoA + 2 NAD^+
r_8	:	Acetyl-CoA + ADP + P_i + 0.018 H^+	$\longrightarrow$	Acetate + CoA + ATP
r_9	:	2 Acetyl-CoA + NADH + H^+	$\longrightarrow$	Crotonyl-CoA + CoA + NAD^+ + H_2O
r_{10}	:	Crotonyl-CoA + 2 NADH + Fd	$\longrightarrow$	Butyryl-CoA + Fd^{2-} + 2 NAD^+
r_{11}	:	Crotonyl-CoA + NADH	$\longrightarrow$	Butyryl-CoA + Fd^{2-}
r_{12}	:	Butyryl-CoA + 2 NADH + 2 H^+	$\longrightarrow$	Butanol + CoA + 2 NAD^+
r_{13}	:	Butyryl-CoA + ADP + P_i + 0.02 H^+	$\longrightarrow$	Butyrate + CoA + ATP
r_{14}	:	ADP + P_i + 4 H^+ (ex)	$\longrightarrow$	ATP + 4 H^+
r_{15}	:	ATP	$\longrightarrow$	ADP + P_i
r_{16}	:	1.33 Glycerol + 11.67 ATP + NH_3 + 1.34 NAD^+	$\longrightarrow$	1 Biomass + 11.67 ADP + 11.67 P_i + 1.34 NADH + 1.34 H^+ + 2 H_2O
r_{17}	:	Fd^{2-} + 2 H^+	$\longrightarrow$	H_2
r_{18}	:	1,3-Propanediol	$\longrightarrow$	1,3-Propanediol (ex)
r_{19}	:	Lactate	$\longrightarrow$	Lactate (ex)
r_{20}	:	Formate	$\longrightarrow$	Formate (ex)
r_{21}	:	Ethanol	$\longrightarrow$	Ethanol (ex)
r_{22}	:	Acetate	$\longrightarrow$	Acetate (ex)
r_{23}	:	Butanol	$\longrightarrow$	Butanol (ex)
r_{24}	:	Butyrate	$\longrightarrow$	Butyrate (ex)
r_{25}	:	H_2	$\longrightarrow$	H_2 (ex)
r_{26}	:	CO_2	$\longrightarrow$	CO_2 (ex)
r_{27}	:	NH_3	$\longrightarrow$	NH_3 (ex)
r_{28}	:	H_2O	$\longrightarrow$	H_2O (ex)
r_{29}	:	Biomass	$\longrightarrow$	

References

Buckel, W., Thauer, R.K., 2018. Flavin-Based Electron Bifurcation, Ferredoxin, Flavodoxin, and Anaerobic Respiration With Protons (Ech) or NAD+ (Rnf) as Electron Acceptors: A Historical Review. Frontiers in microbiology 9, 3704. https://doi.org/10.3389/fmicb.2018.00401.

CHEN, X., SUN, Y., XIU, Z., LI, X., ZHANG, D., 2006. Stoichiometric analysis of biological hydrogen production by fermentative bacteria. International Journal of Hydrogen Energy 31, 539–549. https://doi.org/10.1016/j.ijhydene.2005.03.013.

Lin, D.-S., Yen, H.-W., Kao, W.-C., Cheng, C.-L., Chen, W.-M., Huang, C.-C., Chang, J.-S., 2015. Bio-butanol production from glycerol with Clostridium pasteurianum CH4: the effects of butyrate addition and in situ butanol removal via membrane distillation. Biotechnol Biofuels 8, 168. https://doi.org/10.1186/s13068-015-0352-6.

McAnulty, M.J., Yen, J.Y., Freedman, B.G., Senger, R.S., 2012. Genome-scale modeling using flux ratio constraints to enable metabolic engineering of clostridial metabolism in silico. BMC Systems Biology 6, 42. https://doi.org/10.1186/1752-0509-6-42.

Müller, V., Chowdhury, N.P., Basen, M., 2018. Electron Bifurcation: A Long-Hidden Energy-Coupling Mechanism. Annual Review of Microbiology 72, 331–353. https://doi.org/10.1146/annurev-micro-090816-093440.

Valgepea, K., Loi, K.Q., Behrendorff, J.B., Lemgruber, Renato de S. P., Plan, M., Hodson, M.P., Köpke, M., Nielsen, L.K., Marcellin, E., 2017. Arginine deiminase pathway provides ATP and boosts growth of the gas-fermenting acetogen Clostridium autoethanogenum. Metabolic Engineering 41, 202–211. https://doi.org/10.1016/j.ymben.2017.04.007.

Yoo, M., Bestel-Corre, G., Croux, C., Riviere, A., Meynial-Salles, I., Soucaille, P., 2015. A Quantitative System-Scale Characterization of the Metabolism of Clostridium acetobutylicum. mBio 6, e01808-15. https://doi.org/10.1128/mBio.01808-15.

Zeng, A.-P., Biebl, H., Deckwer, W.-D., 1997. Microbial Conversion of Glycerol to 1,3-Propanediol: Recent Progress, in: Saha, B.C., Woodward, J. (Eds.), Fuels and chemicals from biomass: Developed from a symposium sponsored by the Division of Biochemical Technology [at the 211th National Meeting of the American Chemical Society, New Orleans, Louisiana, March 24 - 28, 1996, vol. 666. American Chemical Society, Washington, DC, pp. 264–279.
