# Supplementary material for: Control of redox potential in a novel continuous bioelectrochemical system led to remarkable metabolic and energetic responses of Clostridium pasteurianum grown on glycerol
Source: Microb Cell Fact. 2022 Sep 1;21:178. doi: 10.1186/s12934-022-01902-5 (PMC9434860; doi:10.1186/s12934-022-01902-5)
Supplement: Supplementary file 2 — Additional file 2: Table S2.1. Estimation of elasticities from kinetic literature data and measured metabolites in C. pasteurianum. Table S2.2. Calculated flux and concentration control coefficients of C. pasteurianum. [file 12934_2022_1902_MOESM2_ESM.pdf]

Supplementary S2

Control of redox potential in a novel continuous bioelectrochemical system led to remarkable metabolic and energetic responses of *Clostridium pasteurianum* grown on glycerol: Metabolic control and regulations analysis

Philipp Arbter, Niklas Widderich, Tyll Utesch, Yaeseong Hong and An-Ping Zeng*

Institute of Bioprocess and Biosystems Engineering, Hamburg University of Technology, Denickestraße 15, D-21073 Hamburg, Germany

*corresponding author

Notes and explanations on Table S2.1:

- Since K_s-values for J_1, J_2, J_5, J_6, J_7, J_8 and J_9 were not available for *C. pasteurianum*, values for *C. acetobutylicum* were taken from the work of Shinto et al. (2007) and Li et al. (2011).
- K_s-values for J_3 (PFOR) and J_4 (PFL) were taken from Moulis et al. (1996) and Thauer et al. (1972).
- Conversion of metabolite levels (c_s) from µmol g^{-1} to mM was done by estimating the intracellular volume of *C. pasteurianum* in relation to the H_2-production rate, as presented in the work of Riebeling et al. (1975).
- As in the work of Li et al. (2011), simple one substrate Michaelis-Menten kinetics were assumed for the blocks. Therefore, elasticities could be obtained by:

$$\varepsilon = \frac{K_s}{K_s + c_s}$$

Table S2.1: Estimation of elasticities from kinetic literature data and measured metabolites in *C. pasteurianum*.

ORP, mV	Block	Substrate	K_s, Mm	c_s, µmol g^{-1}	c_s, mM	Elasticity ε
**-462**	J_1	G3P	26.50	1.09	2.29	0.92
	J_2	pyruvate	500.00	5.83	12.25	0.98
	J_3	pyruvate	0.80	5.83	12.25	0.06
	J_4	pyruvate	1.60	5.83	12.25	0.12
	J_5	acetyl-CoA	30.00	1.72	3.62	0.89
	J_6	acetyl-CoA	51.00	1.72	3.62	0.93
	J_7	acetyl-CoA	1.00	1.72	3.62	0.22
	J_8	butyryl-CoA	5.00	0.33	0.68	0.88
	J_9	butyryl-CoA	6.10	0.33	0.68	0.90
**-416**	J_1	G3P	26.50	2.14	4.48	0.86
	J_2	pyruvate	500.00	3.95	8.31	0.98
	J_3	pyruvate	0.80	3.95	8.31	0.09
	J_4	pyruvate	1.60	3.95	8.31	0.16
	J_5	acetyl-CoA	30.00	3.28	6.89	0.81
	J_6	acetyl-CoA	51.00	3.28	6.89	0.88
	J_7	acetyl-CoA	1.00	3.28	6.89	0.13
	J_8	butyryl-CoA	5.00	0.49	1.03	0.83
	J_9	butyryl-CoA	6.10	0.49	1.03	0.85
**-337**	J_1	G3P	26.50	2.67	6.41	0.81
	J_2	pyruvate	500.00	8.43	20.24	0.96
	J_3	pyruvate	0.80	8.43	20.24	0.04
	J_4	pyruvate	1.60	8.43	20.24	0.07
	J_5	acetyl-CoA	30.00	5.04	12.09	0.71
	J_6	acetyl-CoA	51.00	5.04	12.09	0.81
	J_7	acetyl-CoA	1.00	5.04	12.09	0.08
	J_8	butyryl-CoA	5.00	0.62	1.48	0.77
	J_9	butyryl-CoA	6.10	0.62	1.48	0.81
**-250**	J_1	G3P	26.50	1.06	3.38	0.89
	J_2	pyruvate	500.00	29.09	93.10	0.84
	J_3	pyruvate	0.80	29.09	93.10	0.01
	J_4	pyruvate	1.60	29.09	93.10	0.02
	J_5	acetyl-CoA	30.00	1.06	3.39	0.90
	J_6	acetyl-CoA	51.00	1.06	3.39	0.94
	J_7	acetyl-CoA	1.00	1.06	3.39	0.23
	J_8	butyryl-CoA	5.00	0.07	0.21	0.96
	J_9	butyryl-CoA	6.10	0.07	0.21	0.97

Table S2.2: Calculated flux and concentration control coefficients of *C. pasteurianum*.

			***i* =**										
		C_i^a	**0**	**1**	**2**	**3**	**4**	**5**	**6**	**7**	**8**	**9**	$\sum C_i^a$
		**0**	1.00	0.00	0.00	0.00	0.00	0.00	0.00	0.00	0.00	0.00	1.00
		**1**	1.00	0.00	0.00	0.00	0.00	0.00	0.00	0.00	0.00	0.00	1.00
		**2**	15.74	0.00	1.00	-15.15	-0.59	0.00	0.00	0.00	0.00	0.00	1.00
		**3**	0.96	0.00	0.00	0.07	-0.04	0.00	0.00	0.00	0.00	0.00	1.00
		**4**	1.93	0.00	0.00	-1.86	0.93	0.00	0.00	0.00	0.00	0.00	1.00
		**5**	3.41	0.00	0.00	0.00	0.00	0.86	-0.07	-3.20	0.00	0.00	1.00
**-462 mV**	***a* =**	**6**	3.56	0.00	0.00	0.00	0.00	-0.14	0.93	-3.34	0.00	0.00	1.00
		**7**	0.84	0.00	0.00	0.00	0.00	-0.03	-0.02	0.21	0.00	0.00	1.00
		**8**	0.84	0.00	0.00	0.00	0.00	-0.03	-0.02	0.21	0.17	-0.16	1.00
		**9**	0.86	0.00	0.00	0.00	0.00	-0.03	-0.02	0.21	-0.85	0.83	1.00
		**G3P**	1.09	-1.09	0.00	0.00	0.00	0.00	0.00	0.00	0.00	0.00	0.00
		**PYR**	16.06	0.00	0.00	-15.46	-0.60	0.00	0.00	0.00	0.00	0.00	0.00
		**AcCoA**	3.83	0.00	0.00	0.00	0.00	-0.15	-0.08	-3.60	0.00	0.00	0.00
		**ButyCoA**	0.95	0.00	0.00	0.00	0.00	-0.04	-0.02	0.24	-0.95	-0.19	0.00
		**0**	1.00	0.00	0.00	0.00	0.00	0.00	0.00	0.00	0.00	0.00	1.00
		**1**	1.00	0.00	0.00	0.00	0.00	0.00	0.00	0.00	0.00	0.00	1.00
		**2**	7.32	0.00	0.69	-6.40	-0.61	0.00	0.00	0.00	0.00	0.00	1.00
		**3**	0.67	0.00	-0.03	0.41	-0.06	0.00	0.00	0.00	0.00	0.00	1.00
		**4**	1.20	0.00	-0.05	-1.04	0.90	0.00	0.00	0.00	0.00	0.00	1.00
		**5**	3.09	0.00	-0.13	1.23	0.12	0.77	-0.13	-3.95	0.00	0.00	1.00
**-416 mV**	***a* =**	**6**	3.36	0.00	-0.14	1.33	0.13	-0.25	0.86	-4.29	0.00	0.00	1.00
		**7**	0.50	0.00	-0.02	0.20	0.02	-0.04	-0.02	0.37	0.00	0.00	1.00
		**8**	0.50	0.00	-0.02	0.20	0.02	-0.04	-0.02	0.37	0.04	-0.04	1.00
		**9**	0.51	0.00	-0.02	0.20	0.02	-0.04	-0.02	0.37	-0.98	0.96	1.00
		**G3P**	1.16	-1.16	0.00	0.00	0.00	0.00	0.00	0.00	0.00	0.00	0.00
		**PYR**	7.47	0.00	-0.32	-6.53	-0.62	0.00	0.00	0.00	0.00	0.00	0.00
		**AcCoA**	3.82	0.00	-0.16	1.52	0.14	-0.28	-0.15	-4.88	0.00	0.00	0.00
		**ButyCoA**	0.60	0.00	-0.03	0.24	0.02	-0.04	-0.02	0.44	-1.16	-0.05	0.00
		**0**	1.00	0.00	0.00	0.00	0.00	0.00	0.00	0.00	0.00	0.00	1.00
		**1**	1.00	0.00	0.00	0.00	0.00	0.00	0.00	0.00	0.00	0.00	1.00
		**2**	5.88	0.00	0.23	-4.44	-0.67	0.00	0.00	0.00	0.00	0.00	1.00
		**3**	0.25	0.00	-0.03	0.81	-0.03	0.00	0.00	0.00	0.00	0.00	1.00
		**4**	0.43	0.00	-0.06	-0.32	0.95	0.00	0.00	0.00	0.00	0.00	1.00
		**5**	1.21	0.00	-0.16	2.98	0.45	0.35	0.08	-3.91	0.00	0.00	1.00
**-337 mV**	***a* =**	**6**	1.38	0.00	-0.18	3.40	0.51	-0.75	1.09	-4.46	0.00	0.00	1.00
		**7**	0.14	0.00	-0.02	0.34	0.05	-0.07	0.01	0.56	0.00	0.00	1.00
		**8**	0.14	0.00	-0.02	0.33	0.05	-0.07	0.01	0.56	0.10	-0.10	1.00
		**9**	0.14	0.00	-0.02	0.35	0.05	-0.08	0.01	0.59	-0.94	0.90	1.00
		**G3P**	1.23	-1.23	0.00	0.00	0.00	0.00	0.00	0.00	0.00	0.00	0.00
		**PYR**	6.13	0.00	-0.80	-4.63	-0.70	0.00	0.00	0.00	0.00	0.00	0.00
		**AcCoA**	1.70	0.00	-0.22	4.20	0.64	-0.92	0.12	-5.51	0.00	0.00	0.00
		**ButyCoA**	0.18	0.00	-0.02	0.43	0.07	-0.10	0.01	0.72	-1.16	-0.13	0.00
		**0**	1.00	0.00	0.00	0.00	0.00	0.00	0.00	0.00	0.00	0.00	1.00
		**1**	1.00	0.00	0.00	0.00	0.00	0.00	0.00	0.00	0.00	0.00	1.00
		**2**	2.43	0.00	0.02	-1.45	0.00	0.00	0.00	0.00	0.00	0.00	1.00
		**3**	0.03	0.00	-0.01	0.98	0.00	0.00	0.00	0.00	0.00	0.00	1.00
		**4**	0.06	0.00	-0.02	-0.03	1.00	0.00	0.00	0.00	0.00	0.00	1.00
		**5**	0.05	0.00	-0.02	1.79	0.00	0.28	0.00	-1.10	0.00	0.00	1.00
**-250 mV**	***a* =**	**6**	0.06	0.00	-0.02	1.87	0.00	-0.75	1.00	-1.15	0.00	0.00	1.00
		**7**	0.01	0.00	-0.01	0.46	0.00	-0.18	0.00	0.72	0.00	0.00	1.00
		**8**	0.01	0.00	-0.01	0.46	0.00	-0.18	0.00	0.72	0.00	0.00	1.00
		**9**	0.01	0.00	-0.01	0.46	0.00	-0.19	0.00	0.73	-1.01	1.00	1.00
		**G3P**	1.12	-1.12	0.00	0.00	0.00	0.00	0.00	0.00	0.00	0.00	0.00
		**PYR**	2.90	0.00	-1.17	-1.73	0.00	0.00	0.00	0.00	0.00	0.00	0.00
		**AcCoA**	0.06	0.00	-0.02	1.99	0.00	-0.80	0.00	-1.22	0.00	0.00	0.00
		**ButyCoA**	0.01	0.00	-0.01	0.48	0.00	-0.19	0.00	0.75	-1.04	0.00	0.00

References

Li, R.-D., Li, Y.-Y., Lu, L.-Y., Ren, C., Li, Y.-X., Liu, L., 2011. An improved kinetic model for the acetone-butanol-ethanol pathway of Clostridium acetobutylicum and model-based perturbation analysis. BMC Systems Biology 5, S12. https://doi.org/10.1186/1752-0509-5-S1-S12.

Moulis, J.-M., Davasse, V., Meyer, J., Gaillard, J., 1996. Molecular mechanism of pyruvate-ferredoxin oxidoreductases based on data obtained with the Clostridium pasteurianum enzyme. FEBS Letters 380, 287–290. https://doi.org/10.1016/0014-5793(96)00062-2.

Riebeling, V., Jungermann, K., Thauer, R.K., 1975. The Internal-Alkaline pH Gradient, Sensitive to Uncoupler and ATPase Inhibitor, in Growing Clostridium pasteurianum. European Journal of Biochemistry 55, 445–453. https://doi.org/10.1111/j.1432-1033.1975.tb02181.x.

Shinto, H., Tashiro, Y., Yamashita, M., Kobayashi, G., Sekiguchi, T., Hanai, T., Kuriya, Y., Okamoto, M., Sonomoto, K., 2007. Kinetic modeling and sensitivity analysis of acetone-butanol-ethanol production. Journal of Biotechnology 131, 45–56. https://doi.org/10.1016/j.jbiotec.2007.05.005.

Thauer, R.K., Kirchniawy, F.H., Jungermann, K.A., 1972. Properties and Function of the Pyruvate-Formate-Lyase Reaction in Clostridiae. European Journal of Biochemistry 27, 282–290. https://doi.org/10.1111/j.1432-1033.1972.tb01837.x.
